# Dissecting Drug-Induced Cytotoxicity and Metabolic Dysfunction in Conditionally Immortalized Human Proximal Tubule Cells

**DOI:** 10.3389/ftox.2022.842396

**Published:** 2022-02-28

**Authors:** Charlotte A. Hoogstraten, Jan A. M. Smeitink, Frans G. M. Russel, Tom J. J. Schirris

**Affiliations:** ^1^ Department of Pharmacology and Toxicology, Radboud Institute for Molecular Life Sciences, Radboud University Medical Center, Nijmegen, Netherlands; ^2^ Radboud Center for Mitochondrial Medicine, Radboud University Medical Center, Nijmegen, Netherlands; ^3^ Department of Pediatrics, Radboud University Medical Center, Nijmegen, Netherlands; ^4^ Khondrion BV, Nijmegen, Netherlands

**Keywords:** acute kidney injury, drug-induced toxicity, cellular metabolic activity, cell viability, metabolic toxicity

## Abstract

Fourteen to 26 percent of all hospitalized cases of acute kidney injury are explained by drug-induced toxicity, emphasizing the importance of proper strategies to pre-clinically assess renal toxicity. The MTT assay is widely used as a measure of cell viability, but largely depends on cellular metabolic activity. Consequently, MTT as a single assay may not be the best way to assess cytotoxicity of compounds that reduce mitochondrial function and cellular metabolic activity without directly affecting cell viability. Accordingly, we aim to highlight the limitations of MTT alone in assessing renal toxicity of compounds that interfere with metabolic activity. Therefore, we compared toxic effects observed by MTT with a fluorescent assay that determines compromised plasma membrane permeability. Exposure of proximal tubule epithelial cells to nephrotoxic compounds reduced cellular metabolic activity concentration- and time-dependently. We show that compared to our fluorescence-based approach, assessment of cellular metabolic activity by means of MTT provides a composite readout of cell death and metabolic impairment. An approach independent of cellular metabolism is thus preferable when assessing cytotoxicity of compounds that induce metabolic dysfunction. Moreover, combining both assays during drug development enables a first discrimination between compounds having a direct or indirect mitochondrial toxic potential.

## Introduction

A steadily increasing number of commonly used drugs as well as a large number of experimental compounds have been associated with acute kidney injury. It is a major disease burden, affecting 20 percent of hospitalized adults worldwide (Siew and Davenport, 2015; Ishimoto and Inagi, 2016). Mortality rates are over 50 percent in severe cases and less severe manifestations are associated with chronic kidney disease (Hoste et al., 2015). Various prospective cohort studies indicated that drug-induced mechanisms explain 14 to 26 percent of acute kidney injury cases, emphasizing the importance of renal toxicity (Mehta et al., 2004; Moffett and Goldstein, 2011; Perazella, 2018). Early detection of drug-induced toxicity, and particularly renal toxicity, is also an essential part of lead-optimization in drug development, as it influences the success of a candidate compound to proceed in the developmental process. However, lowering the high drug attrition rates in clinical drug development phases remains a key challenge for pharmaceutical companies. Despite the reduction in drug candidate failures due to poor pharmacokinetic profiles, issues regarding efficacy and safety still lead to high attrition rates (Kola and Landis, 2004; Bunnage, 2011; Hay et al., 2014; Waring et al., 2015). This has been confirmed by an inventory of the main reasons for drug attrition from four leading pharmaceutical companies, showing a substantial contribution of safety-related (*i.e.,* non-clinical toxicology) causes (Waring et al., 2015). Especially nephrotoxicity is often observed in clinical studies that was not detected earlier during non-clinical development (Troth et al., 2019). Hence, improved non-clinical testing of therapeutic candidates for their nephrotoxic potential is warranted to reduce attrition rates in clinical development phases (van Tonder et al., 2015).

**FIGURE 1:**
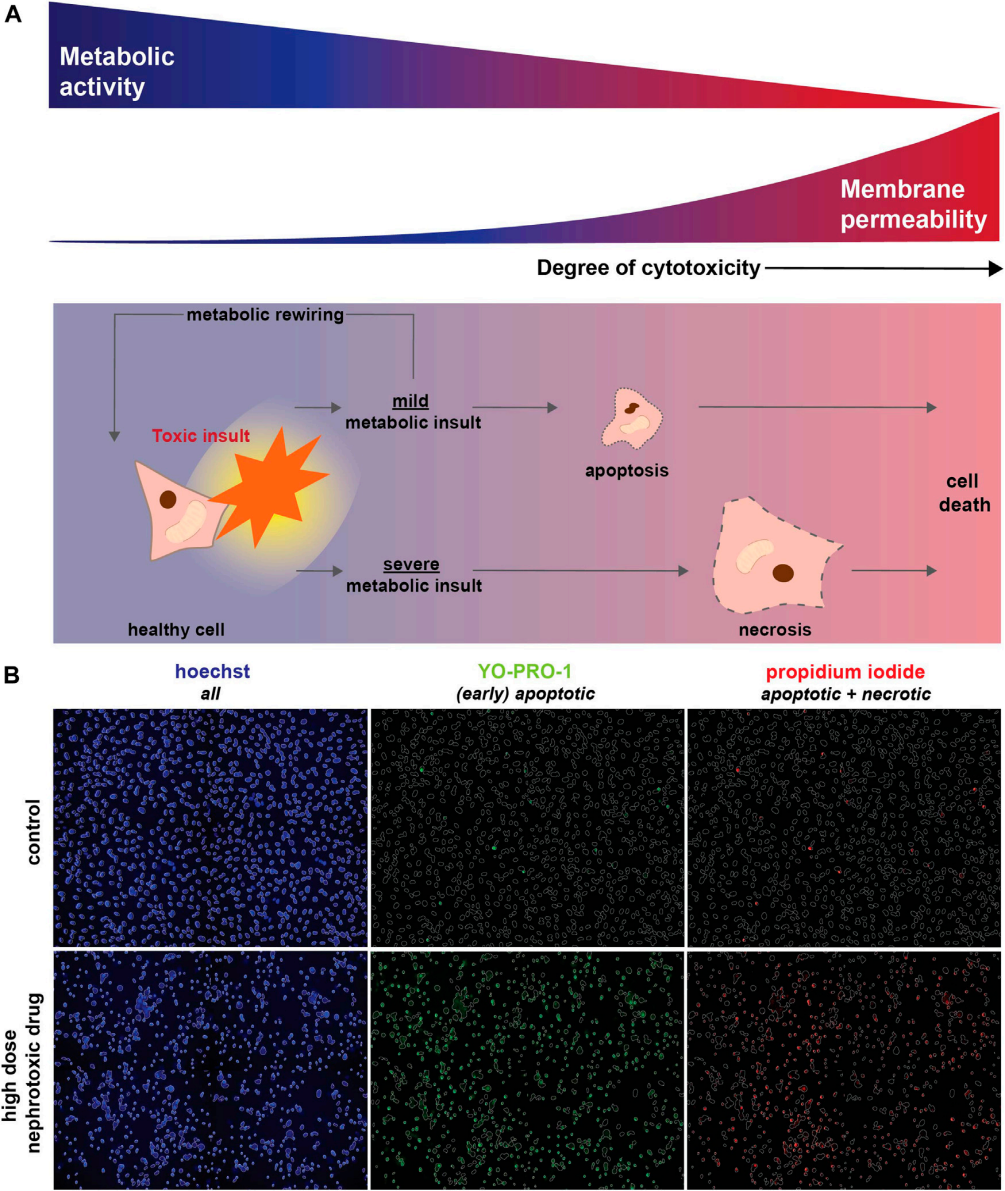
Toxic (metabolic) insults interfere with cellular metabolic activity and eventually result in cell death. **(A)** Upon a toxic insult, cellular metabolism could be only mildly reduced leading to the induction of apoptosis. Alternatively, cellular viability might be restored via metabolic rewiring (*i.e.*, the capacity of cells to switch to alternative metabolic pathways upon changing conditions, including nutrient availability or drug exposure). However, when a toxic insult severely inhibits metabolic function it triggers necrosis, which results in loss of membrane integrity and ultimately cell death. As MTT measures cellular metabolic activity, this method will also detect changes in case of a mild toxic insult, which may eventually not affect cell viability (blue). In contrast, a fluorescent-based cell death detection approach based on membrane permeability will only be relevant if apoptotic or necrotic mechanisms have been activated (red). **(B)** Fluorescent-based analysis of cell death combines hoechst (blue) to visualize all cells with Yo-Pro-1-iodide (green) that crosses plasma membranes of (early) apoptotic cells in particular and propidium iodide (red) that stains necrotic cells. Cell death, detected as Yo-Pro-1-iodide and propidium iodide positive cells, is induced by a high concentration of a nephrotoxic drug (cisplatin, 100 μM) compared to a vehicle control.

Obtaining accurate and reliable results from *in vitro* cytotoxicity assays is therefore of vital importance. In the 1980s, Mosmann developed a cell viability and proliferation assay, measuring the reductive activity as enzymatic conversion of 3-(4,5-dimethylthiazol-2-yl)-2,5-diphenyl-2H-tetrazolium bromide (MTT) into dimethyl sulfoxide (DMSO)-soluble formazan crystals by dehydrogenases, which is nowadays often recognized as the “golden standard” for cytotoxicity assessment (van Tonder et al., 2015; Mosmann, 1983). However, the MTT assay determines cellular metabolic activity, as it mainly relies on the activity of mitochondrial dehydrogenases, in particular succinate dehydrogenase (Rai et al., 2018). Consequently, this method may not be suitable to distinguish drug-induced metabolic suppression from reduced cell viability, as a reduced cellular metabolic activity does not necessarily lead to the latter (Figure 1A). In this respect, cellular metabolic rewiring may spare a cell from entering cell death pathways, but still demonstrate a reduced mitochondrial dehydrogenase activity and hence reduced the MTT signal (Figure 1A). This limitation of MTT has been described for various known inhibitors of cellular metabolism in three human breast carcinoma cell lines (van Tonder et al., 2015). Similarly, radiation-induced cellular metabolic hyperactivation biases the ability of the MTT assay to assess cell viability (Rai et al., 2018). Moreover, MTT reduction can be influenced by other enzymes linked with cellular redox status, like glutathione S-transferases, further warranting cautious interpretation of results obtained with MTT using compounds that reduce cellular metabolism (York et al., 1998). Finally, chemical reduction of MTT has been found in other organelles such as endoplasmic reticulum and may also explain MTT reduction by flavonoids and polyphenols (Pagliacci et al., 1993; Peng et al., 2005; Wang et al., 2010; Stockert et al., 2012; van Tonder et al., 2015).

Similar limitations of the MTT assay may also be relevant for the assessment of renal toxicity, as mitochondrial dysfunction is described for a vast number of nephrotoxic drugs (Sanz et al., 2018). Proximal tubule cells, which are most often affected in drug-induced renal toxicity, are metabolically very active and have a high mitochondrial content (Bhargava and Schnellmann, 2017). It has, however, not been investigated whether MTT is the most suitable assay to assess the nephrotoxic potential of drugs. Therefore, we compared the MTT assay with the determination of cell viability by a method independent of cell metabolism, which is based on cell membrane permeability (Gawlitta et al., 2004).

Exposing renal proximal tubule cells to a selection of prototypic nephrotoxic drugs in a concentration- and time-dependent manner, we demonstrate that, compared to the fluorescence-based approach, assessment of cellular metabolic activity by means of MTT does not exclusively assess cellular toxicity. Consequently, MTT rather provides a composite readout of cell death and decreased cellular metabolic rate.

## Materials and Methods

### Compounds

Cisplatin and chloroacetaldehyde solution were purchased from Sigma-Aldrich (Zwijndrecht, Netherlands). Sanguinarine (chloride) was from Cayman Chemical (Ann Arbor, MI, United States) and tenofovir was obtained from Santa Cruz Biotechnologies, Inc. (Santa Cruz, CA, United States).

### Cell Culture

To investigate the effects of selected nephrotoxic compounds on proximal tubule cell metabolism and cell viability, human conditionally immortalized proximal tubule epithelial cells expressing organic anion transporter 1 (ciPTEC-OAT1, RRID:CVCL_LI01) were used. OAT1 is expressed at the basolateral membrane of proximal tubule cells and known to import anionic drugs, including tenofovir used in this study (Kohler et al., 2011). As this transporter is not naturally present in ciPTEC cells, it was introduced via stable transfection, described by Wilmer *et al.* (Wilmer et al., 2010). As such, this model is currently the most clinically relevant model available to study pharmacological and toxicological responses that closely resemble human renal physiology *in vitro.* Cells were collected from mid-stream urine of healthy volunteers and immortalized by infection with SV40T and hTERT vectors, as described before (Wilmer et al., 2010; Nieskens et al., 2016; Vriend et al., 2019). Proliferating cells were cultured at 33°C and 5% (*v/v*) CO_2_ at GMO level II safety and hygiene conditions. Medium, consisting of 1:1 Dulbecco’s modified Eagle’s medium and nutrient mixture F-12 without phenol red (DMEM Ham’s F-12, Life Technologies, Paisley, UK), supplemented with 5 μg/ml insulin, 5 μg/ml transferrin, 5 ng/ml selenium, 36 ng/ml hydrocortisone, 10 ng/ml human epidermal growth factor (EGF), 40 pg/ml trio-iodothyrine (all purchased from Sigma-Aldrich), 1% (*v/v*) penicillin/streptomycin (Life Technologies) and 10% (*v/v*) fetal bovine serum (FBS, Greiner Bio-One, Alpen a/d Rijn, Netherlands), further referred to as PTEC complete medium, was refreshed every 2–3 days. Cells used for experiments varied from passage numbers 49 to 58. ciPTEC-OAT1 was seeded in black or transparent/clear flat bottom 96-wells plates at a density of 63,000 cells/cm^2^. Cells proliferated for 1 day at 33°C and 5% (*v/v*) CO_2_ in PTEC complete medium, followed by 7 days maturation at 37°C, 5% (*v/v*) CO_2_ in PTEC complete medium without antibiotics to differentiate into an epithelial monolayer. Mature ciPTEC-OAT1 were exposed to prototypic nephrotoxic compounds, including cisplatin, tenofovir, sanguinarine and chloroacetaldehyde in a serial √10-dilution (0.1–1,000 μM, unless stated otherwise, dissolved in PTEC complete medium or DMSO for chloroacetaldehyde), for 0.5, 1, 2, 4, 8, or 24 h at 37°C and 5% (*v/v*) CO_2_, after which cellular metabolic activity or fluorescence-based cell death were assessed. DMSO concentrations did not exceed 0.1% (*v/v*). Determination of IC_70_ and IC_90_ concentrations (Table 1) for time-dependent compound exposure was based on concentration-response curves of analyzed cellular metabolic activity, which were normalized to unexposed ciPTEC-OAT1.

**TABLE 1:** Overview of prototypic nephrotoxicants with calculated IC_70_- and IC_90_-concentrations. IC_70_- and IC_90_-concentrations are defined as inhibitory concentrations that result in 70% and 90% of the maximal inhibitory effect on cellular metabolic activity, respectively. IC_70_ and IC_90_ concentrations were based on and calculated using IC_50_-values of mean cellular metabolic activity concentration-response curve fittings (Figure 1). 95% confidence bands were plotted in curve fitting graphs and used to calculate confidence intervals (CI) for IC_70_ and IC_90_-concentrations.

Compound	IC_70_ (µM)	IC_90_ (µM)
	*(95%–CI)*	*(95%–CI)*
Chloroacetaldehyde	22 (*17–28*)	36 (*25–59*)
Cisplatin	28 (*18–38*)	43 (*28–∞*)
Sanguinarine	4 (*3–5*)	11 (*6–∞*)
Tenofovir	231 (*135–427*)	422 (*182–∞*)

### Analysis of Cellular Metabolic Activity

To evaluate compound-induced effects on cellular metabolic activity in ciPTEC-OAT1, a colorimetric assay based on tetrazolium salt MTT (3-(4,5-dimethylthiazol-2-yl)-2,5-diphenyl tetrazolium bromide), was performed (Mosmann, 1983). In short, ciPTEC-OAT1 were cultured and exposed as described above in transparent/clear flat bottom 96-wells plates (Corning, Amsterdam, Netherlands) and compound-exposed ciPTEC-OAT1 were washed three times with serum-free PTEC complete medium, followed by incubation with 0.5 mg/ml MTT in serum-free PTEC medium for 3 h at 37°C and 5% (*v/v*) CO_2_. Formed formazan crystals were dissolved in DMSO (Merck, Darmstadt, Germany) on a microplate shaker (VWR, Radnor, PA, United States) for 2 h. Absorption was measured at 560 nm and subtracted from background at 670 nm using Benchmark (Bio-Rad, Veenendaal, Netherlands). Values were normalized to unexposed ciPTEC-OAT1 control.

### Fluorescence-Based Analysis of Cell Death

ciPTEC-OAT1 were seeded in black/clear flat bottom 96-wells plates (Fisher Scientific, Landsmeer, Netherlands), cultured and concentration- and time-dependently exposed as described above. Nuclei were stained using hoechst 33342 (20 μg/ml, Life Technologies), Yo-Pro-1-iodide (2 μM, Life Technologies) and propidium iodide (1 μg/ml, Sigma-Aldrich) to differentiate all, early apoptotic, and necrotic cells, respectively (Schirris et al., 2015; Vriend et al., 2019). Cells were incubated with a mixture of the dyes for 30 min at 37°C. Fluorescence was imaged at a total ×100 magnification using Becton Dickinson (BD) Pathway 855 high-throughput microscope (BD Bioscience, Breda, Netherlands). Obtained images were analyzed for viable and non-viable cells using CellProfiler™ - 3.0.0 (Carpenter et al., 2006; Kamentsky et al., 2011; McQuin et al., 2018).

### Statistical Analysis

Curve-fitting and statistical data analysis were performed using GraphPad prism v5.03 (GraphPad Software Inc., San Diego, CA). Data were normalized to untreated (vehicle) control ciPTEC-OAT1. A response was considered cytotoxic when a significant reduction in cell viability was observed. Results were plotted after background subtraction using nonlinear regression with four parameters and variable slope, constraining the bottom to greater than 0.0 to prevent a lower limit representing negative cell viability on log-transformed x-values. Statistical significance of concentration- and time-dependent data was determined by one-way ANOVA, followed by Dunnett’s *post hoc* analysis to correct for multiple comparison. Differences between assay types (p_assay_) were statistically analyzed using two-way ANOVA and corrected for multiple comparison by Bonferroni’s *post hoc* analysis. All data is presented as mean ± standard error of mean (SEM) of at least three independent experiments (n = 3), performed with five or six experimental replicates.

## Results

### MTT Assay Shows a Stronger Concentration-Dependent Decrease Upon Exposure to Nephrotoxic Compounds Compared to Fluorescence-Based Cell Death Assessment

Comparison of nephrotoxicity detected by MTT and our fluorescence microscopy method (Figure 1) was performed using four prototypic nephrotoxic drugs, which previously also showed to induce mitochondrial dysfunction. These compounds cover a range of different drug classes and include the antineoplastic drug cisplatin, the antiretroviral drug tenofovir, the Na^+^/K^+^-ATPase inhibitor sanguinarine, and chloroacetaldehyde, the cytotoxic metabolite of the antineoplastic drug ifosfamide. Conditionally immortalized proximal tubule epithelial cells (ciPTEC) expressing the organic anion transporter 1 (ciPTEC-OAT1), were exposed for 24 h to a concentration-range of all four compounds. Fluorescence-based analysis of cell death combines Yo-Pro-1-iodide that specifically stains nuclei of apoptotic cells with the nuclear dye propidium iodide, which only crosses plasma membranes of necrotic cells, and hoechst to visualize nuclei of all cells [Figure 1B (Idziorek et al., 1995; Liu et al., 2004; Boffa et al., 2005; Schirris et al., 2015)]. Hoechst-stained nuclei were used to create a mask of all cells, which was used to quantify the fraction of apoptotic and necrotic cells (Figure 1B).

**FIGURE 2:**
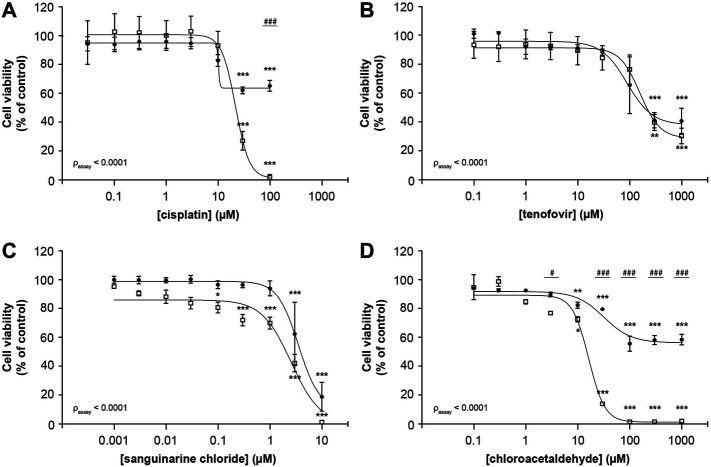
Comparison of MTT and fluorescence-based cell death methods to assess concentration-dependent reduction of ciPTEC cellular viability. Cellular metabolic activity (□) and fluorescence-based cell death (•) were determined in ciPTEC-OAT1 after 24 h incubation with a serial √10-dilution of nephrotoxic compounds **(A)** cisplatin, **(B)** tenofovir, **(C)** sanguinarine chloride and **(D)** chloroacetaldehyde. All results were normalized to unexposed controls. Statistical analyses: one-way ANOVA corrected for multiple comparison by Dunnett’s *post hoc* analysis to compare differences between vehicle control and exposed conditions *p < 0.05, **p < 0.01, ***p < 0.001; and two-way ANOVA corrected for multiple comparison by Bonferroni’s *post hoc* analysis to compare differences between assays ^*#*^ *p* < 0.05, ^*###*^ *p* < 0.001. *p*-value corresponding to overall significance between assays is indicated for each compound. Mean ± SEM; n = 3 independent experiments.

Cisplatin reduced cellular metabolic activity to 1.6 ± 2.0% whereas fluorescence-based cell death only lowered viable cells to 68 ± 4% (100 μM; Figure 2A), suggesting that it substantially impedes cellular metabolic activity in addition to cytotoxicity (p_assay_ < 0.0001). For tenofovir a reduction to 32 ± 6% and 43 ± 9% in viable cells was observed using MTT and fluorescence microscopy, respectively (1,000 µM), which did not significantly differ between both assays (p_assay_ = 0.56; Figure 2B). Due to poor solubility of sanguinarine chloride, concentrations up to only 10 µM could be investigated. Consequently, the concentration-response bottom plateau could not be determined experimentally, but sanguinarine almost completely reduced cell viability, with residual cell viability levels of 1.4 ± 0.1% and 20 ± 10% (10 μM; Figure 2C) using the MTT or fluorescence-based cell death analysis, respectively, with a statistically significant difference between both assays (p_assay_ < 0.0001). At increasing chloroacetaldehyde concentrations cell viability significantly differed when measured by MTT compared to our fluorescence-based cell death (p_assay_ < 0.0001; 2.13 ± 0.05% and 61.0 ± 0.8%, respectively; 1,000 μM; Figure 2D). These observations imply that previously described toxic effects determined using the MTT assay of at least cisplatin, sanguinarine and chloroacetaldehyde, could have been influenced by compound interference with the metabolizing capacity of the cell. Consequently, it emphasizes the essential difference in readout between both assays described and warrants careful interpretation of results.

### Nephrotoxic Compounds Time-Dependently Interfere With Cellular Metabolic Activity and Fluorescence-Based Cell Death

**FIGURE 3:**
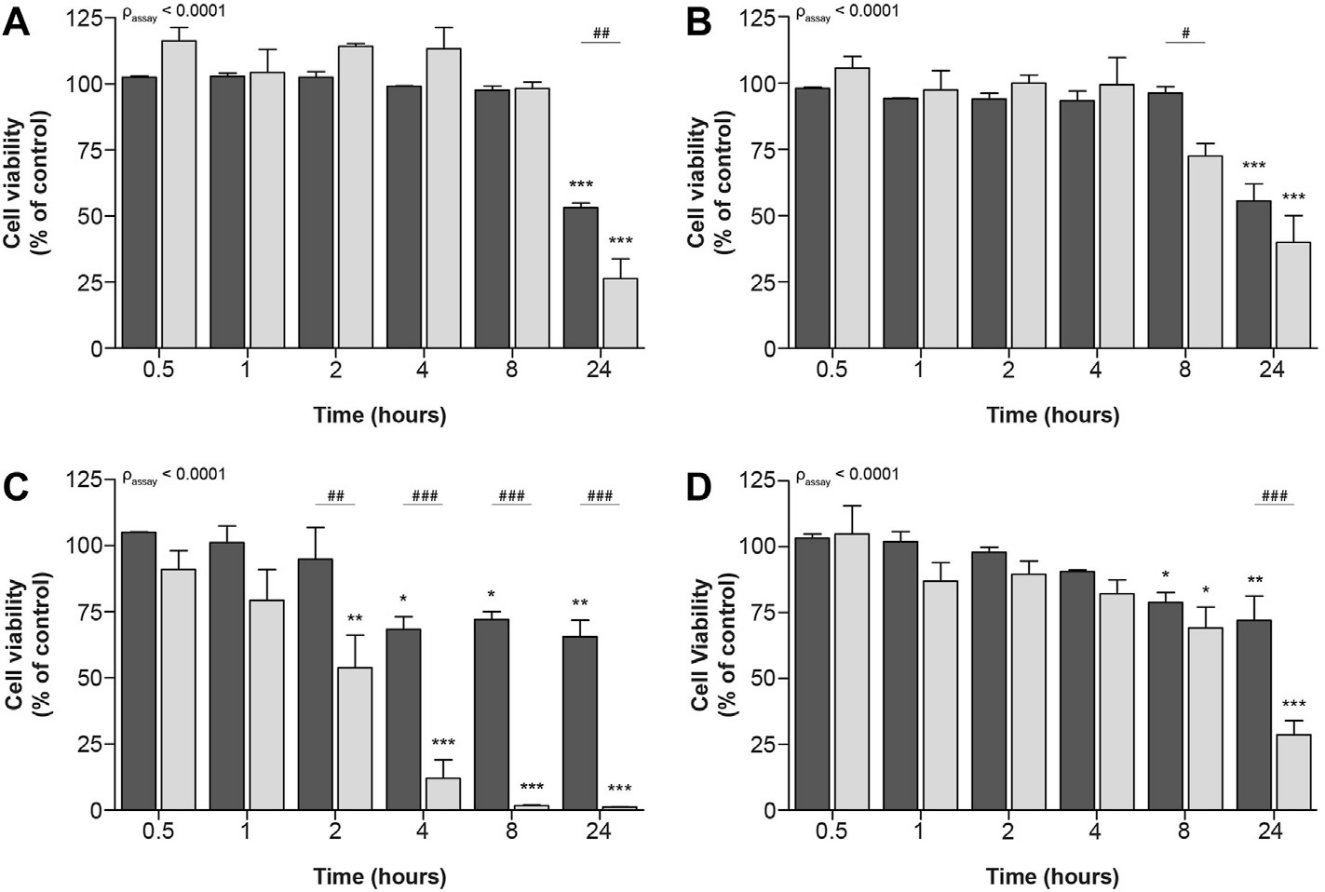
Time-dependent effects upon exposure of ciPTEC-OAT1 to IC_90_-concentrations of nephrotoxic drugs on cellular metabolic activity and fluorescence-based cell death. Mature ciPTEC-OAT1 were exposed to IC_90_-concentrations of nephrotoxic drugs for 0.5, 1, 2, 4, 8 or 24 h, after which fluorescence-based cell death (■) or cellular metabolic activity (■) assays were performed. **(A)** cisplatin, **(B)** tenofovir, **(C)** sanguinarine, **(D)** chloroacetaldehyde. For IC_90_-values see table 1. All results were normalized to unexposed controls. Statistical analyses: one-way ANOVA corrected for multiple comparison by Dunnett’s *post hoc* analysis to compare differences between vehicle control and exposed conditions *p < 0.05, **p < 0.01, ***p < 0.001; and two-way ANOVA corrected for multiple comparison by Bonferroni’s *post hoc* analysis to compare differences between assays ^*#*^ *p* < 0.05, ^*##*^ *p* < 0.01, ^*###*^ *p* < 0.001. *p*-value corresponding to overall significance between assays is indicated for each compound. Mean ± SEM; n = 3 independent experiments.

Remarkably, no differences between both assays could be observed for the prototypic nephrotoxic drug tenofovir. It has, however, been described that drugs can decrease cellular metabolic activity before cellular viability is compromised (Verma et al., 2019). Consequently, we investigated whether at earlier timepoints differences between both methods could show contrasting results for tenofovir and all other compounds. These time-dependent effects were measured after 0.5, 1, 2, 4, 8 and 24 h. To this end, ciPTEC-OAT1 cells were exposed to IC_70_ and IC_90_ drug concentrations (calculated from concentration-dependent results of cellular metabolic capacity, see Table 1), as the commonly used IC_50_ concentration is likely subjected to variation due to its position in the steepest part of the curve. All prototypic nephrotoxicants showed a significant time-dependent reduction in cell viability as measure of cellular metabolic activity and fluorescence-based cell death (Figure 3). For cisplatin and tenofovir (Figures 3A, B, respectively), this effect was only observed 24 h after incubation. Also, both assays significantly differed 24 h after incubation for cisplatin (*p* < 0.01; Figure 3A) and 8 h after incubation for tenofovir (*p* < 0.05; Figure 3B). The most prominent difference was noted for sanguinarine (Figure 3C). A significant reduction in cell viability was observed 2 h after incubation (*p* < 0.0001), whereas a significantly lower cell viability as determined by fluorescence microscopy occurred only 4 h after incubation (*p* < 0.05). Overall, cellular metabolic activity showed a stronger reduction compared to the fluorescence-based approach (p_assay_ < 0.0001), indicating that sanguinarine primarily interferes with metabolic capacity, while cell death originates secondary to a reduced metabolic activity. Remarkably, 8 h after compound exposure, cellular metabolic activity was very low (1.7 ± 0.2%), while the nuclear staining approach showed a residual viability of 72 ± 3%, which could suggest activation of other compensatory cellular metabolic pathways that maintain cells viable. Chloroacetaldehyde showed a reduction in cell viability as measure of both cellular metabolic activity and fluorescence-based cell death, starting 8 h after compound incubation (Figure 3D). After 24 h of incubation, cellular metabolic activity was reduced to a viability of 29 ± 5% (*p* < 0.0001), whereas according to the fluorescence-based assay, cell death decreased to only 72 ± 9% of control (*p* < 0.01).

**FIGURE 4:**
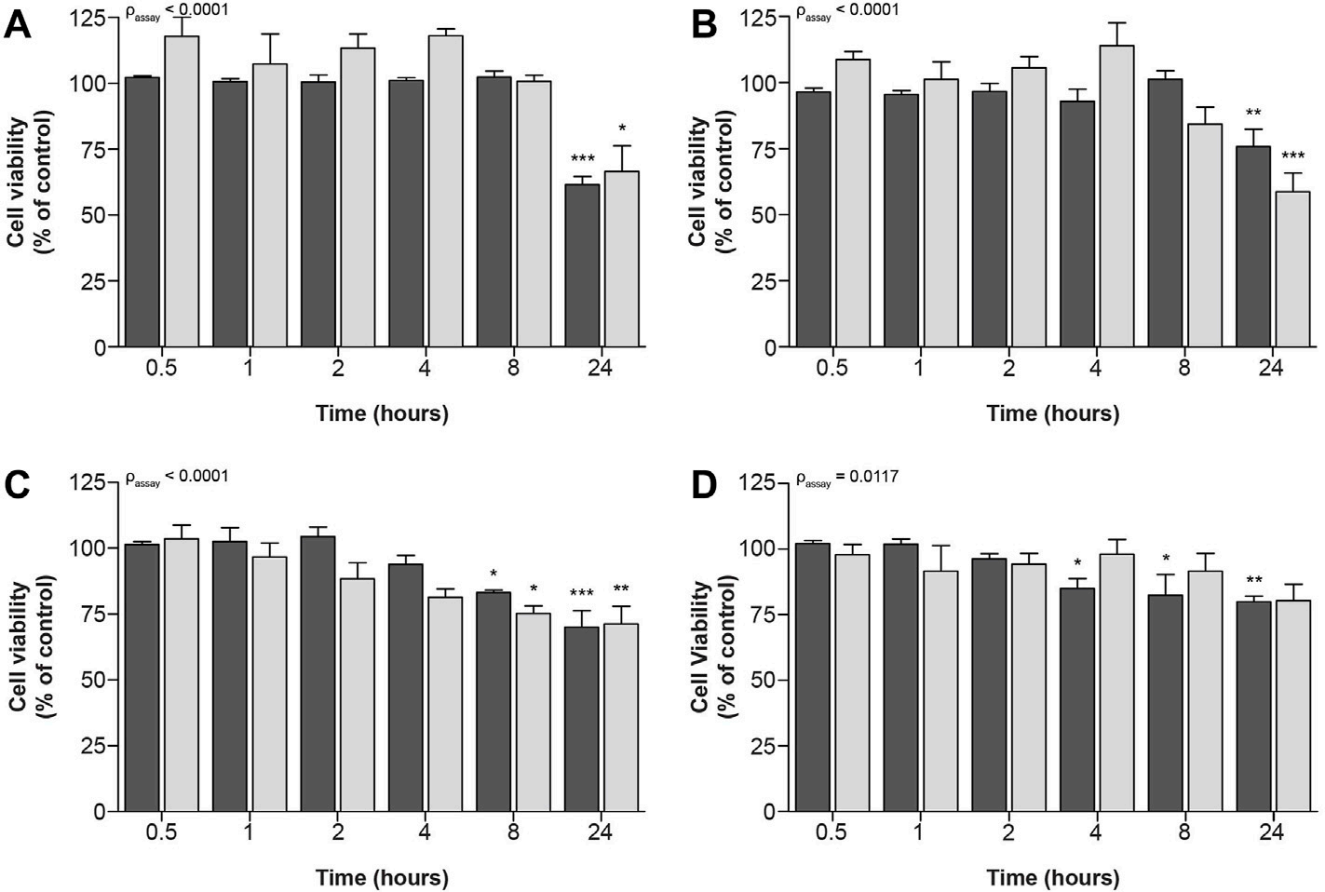
Time-dependent effects upon exposure of ciPTEC-OAT1 to IC_70_-concentrations of nephrotoxic drugs on cellular metabolic activity and fluorescence-based cell death. Mature ciPTEC-OAT1 were exposed to IC_70_ concentrations of nephrotoxic drugs for 0.5, 1, 2, 4, 8 and 24 h, after which fluorescence-based cell death or (■) cellular metabolic activity (■) assays were performed. **(A)** cisplatin, **(B)** tenofovir, **(C)** sanguinarine, **(D)** chloroacetaldehyde. For IC_70_-values see Table 1. All results were normalized to unexposed controls. Statistical analyses: one-way ANOVA corrected for multiple comparison by Dunnett’s *post hoc* analysis to compare differences between vehicle control and exposed conditions *p < 0.05, **p < 0.01, ***p < 0.001; and two-way ANOVA corrected for multiple comparison by Bonferroni’s *post hoc* analysis to compare differences between assays. *p*-value corresponding to overall significance between assays is indicated for each compound. Mean ± SEM; n = 3 independent experiments.

Similar, but less prominent effects of compound exposure on cellular metabolic activity and fluorescence-based cell death could be observed for exposure to IC_70_-concentrations (Figure 4), at which all four compounds significantly decreased cellular viability.

## Discussion

Drug-induced kidney injury is one of the most frequently observed adverse effects and often leads to high attrition rates during clinical drug development stages (Siew and Davenport, 2015; Troth et al., 2019). This warrants the use of proper *in vitro* strategies to assess potential toxic drug effects during early drug development and design. Importantly, many drugs are known to disturb mitochondrial function (Cez et al., 2018), which may bias the interpretation of results obtained by commonly used tetrazolium salt-based methods that rely on enzymatic activity, such as the MTT assay. Here, we compared the toxicity of four nephrotoxic drugs known to inhibit cellular metabolism measured by the MTT assay and a fluorescence-based method, which is not dependent on cellular metabolism, but on membrane permeability. Differences in concentration- and time-dependent toxicity patterns were observed between both assays, in which the selected drugs interfered stronger and earlier with cellular metabolic capacity. This shows that these drugs reduce the MTT signal before any cellular toxicity is observed, which has often been misinterpreted as cytotoxicity. This underlines the vital need for the selection of proper cell viability methods in early drug safety screening.

The observed strong inhibitory effect of cisplatin on cellular metabolic activity has previously been related to apoptosis through mitochondrial pathways, as generation of (mitochondrial) oxidative stress appeared critical in its mechanism of cytotoxicity (Fuertes et al., 2003; Cullen et al., 2007; Marullo et al., 2013; Choi et al., 2015; Yu et al., 2018). A similar strong reduction in the MTT assay has been reported before (Nieskens et al., 2018), and a weaker inhibitory effect using our fluorescence-based cell death assay is in line with the observed cisplatin-induced mitochondrial dysfunction. For tenofovir, a comparable time-dependent effect on cellular metabolic capacity and cytotoxicity was detected. The slightly stronger metabolic response compared to fluorescence-based cell death confirms previous observations that tenofovir targets mitochondria (*e.g.,* ultrastructural defects and mtDNA depletion) of the renal proximal tubule (Lee et al., 2003; Lewis et al., 2003; Kohler et al., 2009; Milián et al., 2017). However, a sole role for mitochondria is not expected, as cellular accumulation through active uptake via OAT1 and decreased efflux into tubular lumen has previously been described as the main mechanism of tenofovir-induced nephrotoxicity (Jafari et al., 2014). Disruption of mitochondrial biogenesis would explain further reduction of mitochondrial enzyme abundance, including dehydrogenases. Sanguinarine also demonstrated a strong reduction in cellular metabolic activity, whereas fluorescence-based cell death was less severely affected. Serious toxicity of sanguinarine has been recognized before (Gong et al., 2018) and time-dependent differences between both assays align with previously observed mitochondrial effects, including depolarization of the mitochondrial membrane potential and inhibition of cellular respiration (Chang et al., 2007; Serafim et al., 2008). Finally, the strong metabolic effects of the ifosfamide metabolite chloroacetaldehyde is in agreement with previously observed toxicity in rabbit renal proximal tubule cells, in which ATP levels were concentration-dependently reduced (Springate et al., 1999). Moreover, chloroacetaldehyde seems to inhibit hexokinase (Knouzy et al., 2010), which further explains the strong cellular metabolic effects observed in our study. Overall, our results are in accordance with previously reported cytotoxicity of the drugs studied, and differences between both assays can be explained by effects of the various nephrotoxicants on cellular metabolism.

The observed strong concentration- and time-dependent patterns in our study are, however, expected not to be specific to the MTT assay. As similar tetrazolium-based assays, including XTT, WST-1/8 and CCK-8, all rely on enzymatic conversion of a substrate by metabolic capacity of the cell (Mosmann, 1983; Ginouves et al., 2014), these are expected to show similar patterns after exposure to metabolically active drugs. Therefore, these enzymatic assays are not preferred in assessing cellular effects of compounds that impair metabolic activity, as they provide a composite readout consisting of cytotoxic and metabolic effects. Besides our fluorescence-based method, other approaches independent of cellular metabolism, including Annexin-V and TUNEL assays have extensively been applied to examine cell death, also in high-throughput settings (Gelles and Chipuk, 2016; Arifulin et al., 2017). Apoptosis detection by both methods is, however, limited as compared to Yo-Pro-1, which makes the latter a more sensitive approach to identify cytotoxicity in drug development (Wlodkowic et al., 2011; Fujisawa et al., 2014).

Our observation that cellular metabolic capacity is time-dependently affected by the drugs evaluated in this study highlights the importance of differentiating between metabolically-dependent and -independent cytotoxicity assays. These observations are especially important for primary mitochondrial toxicants as these compounds are expected to decrease mitochondrial function before cell viability is affected, as we observed for sanguinarine. Interestingly, time-dependent comparison of (nephrotoxic) compounds for their cellular toxicity, could provide an ideal experimental setup to differentiate between primary metabolic active drugs and drugs that inhibit cellular metabolism via indirect mechanisms (secondary mitochondrial toxicants), including inhibitors of other metabolic pathways. Consequently, such a combination of assays may be relevant when screening for (renal) toxicity in early drug development. Moreover, combining methods with different readouts might contribute to the prevention of unnecessary drug attritions. Additionally, differentiating between primary and secondary mitochondrial toxicants is also applicable in pre-clinical studies investigating drug-related adverse effects in other metabolically active tissues such as hepatocytes, enterocytes and muscle cells.

To conclude, methods measuring cellular metabolic activity, like the MTT assay, do not exclusively assess cellular toxicity. They rather provide a composite readout of cell death and decreased metabolic rate, as opposed to cytotoxicity assessed using assays that do not rely on cellular metabolism. Such an assay would be more suitable for assessing toxicity of compounds that interfere with cellular metabolism. A combination of assays is preferred for initial toxicity screening in drug development to discriminate between drugs that do and do not inhibit cellular metabolic activity. Selection of the proper cell viability assessment methods in early drug safety screening or when assessing toxicity of existing drugs is vital, and will contribute to the development of drugs with lower (nephro)toxic potential and improved safety profile.

## Data Availability

The original contributions presented in the study are included in the article/supplementary material, further inquiries can be directed to the corresponding authors.
